# Higenamine in Plants as a Source of Unintentional Doping

**DOI:** 10.3390/plants11030354

**Published:** 2022-01-27

**Authors:** Vanya Rangelov Kozhuharov, Kalin Ivanov, Stanislava Ivanova

**Affiliations:** Department of Pharmacognosy and Pharmaceutical Chemistry, Faculty of Pharmacy, Medical University-Plovdiv, 4002 Plovdiv, Bulgaria; kalin.ivanov@mu-plovdiv.bg (K.I.); stanislava.ivanova@mu-plovdiv.bg (S.I.)

**Keywords:** higenamine in plants, *Nelumbo nucifera*, *Tinospora crispa*, *Nandina domestica*, *Gnetum parvifolium*, *Asarum siebodii*, *Aconitum carmichaelii*, *Aristolochia brasiliensis*

## Abstract

Background: Higenamine is a β_2_ agonist of plant origin. The compound has been included in WADA’s prohibited list since 2017. Higenamine may be detected in different plants and many food supplements of natural origin. Methods: Our literature search was conducted through PubMed, Science Direct, Google Scholar, and Web of Science studies investigating the presence of higenamine in plants that are used in traditional folk medicine or included in food supplements. Our study aimed to assess the risk of adverse analytical findings caused by higenamine-containing plants. Results: Based on our literature search, *Nelumbo nucifera, Tinospora crispa, Nandina domestica, Gnetum parvifolium, Asarum siebodii,* *Asarum heterotropoides*, *Aconitum carmichaelii,* and *Aristolochia brasiliensis* are higenamine-containing plants. Based on data from Eastern folk medicine, these plants can provide numerous health benefits. Professional athletes likely ingest these plants without knowing that they contain higenamine; these herbs are used in treatments for different conditions and various foods/food supplements in addition to folk medicine. Conclusion: Athletes and their teams must be aware of the issues associated with the use of plant-based products. They should avoid consuming higenamine-containing plants during and outside of competition periods.

## 1. Introduction

Plant species are one of the most significant sources for current drug discovery [1]. Many important medicines have plant origins, and there are still thousands of unexplored molecules that have obtained from plants. Nowadays, many plant extracts are included in food supplements. The use of these extracts are often based on data available from folk medicine.

The use of herbal supplements by athletes increased in the last 10 years [2]. Herbal food supplements include extracts from seeds, roots, leaves, berries, or whole plants. These extracts generally contain many phytochemicals, such as polyphenols, carotenoids, alkaloids, flavonoids, glycosides, saponins, lignans, etc., which can potentially enhance athletes’ performance or recovery [3,4].

**Figure 1:**
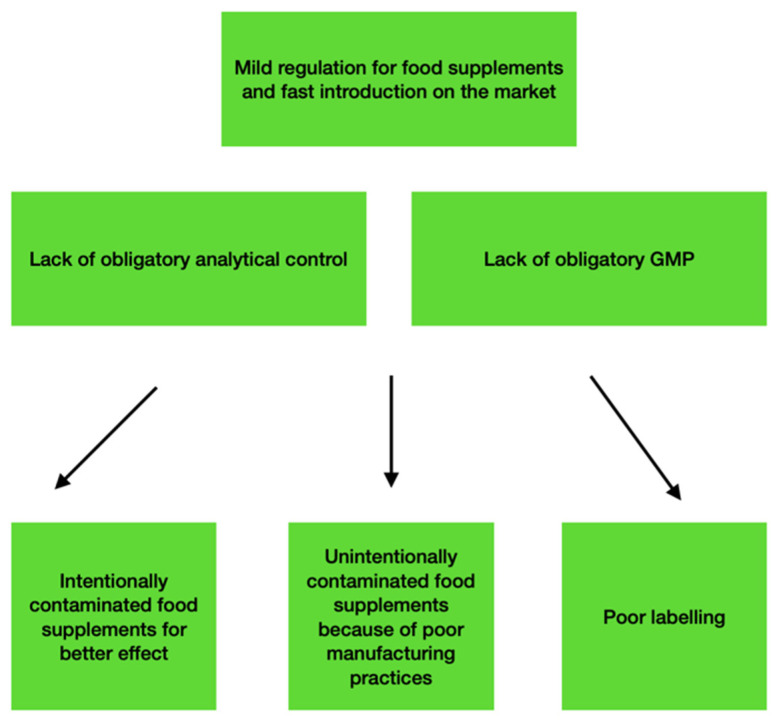
Food supplements as a source of unintentional doping.

Athletes’ complex supplementation is an important part of their nutritional regimes. At the same time, food supplements may be a source of unintentional doping (Figure 1). Many studies have reported on undeclared compounds found in different food supplements (FS) [5,6,7,8,9,10,11,12,13,14,15,16]. The main reasons for the unintentional intake of doping compounds are:

1. Undeclared doping compounds in commercial products are naturally present in the phytochemical composition of plant extracts (e.g., higenamine, ephedrine, synephrine, and morphine).
2. Contaminated food supplements with doping compounds (e.g., anabolic agents, sibutramine, clenbuterol).
3. Insufficient educational programs for doping prevention.

The intake of such products exposes athletes to risk. Usually, FS-containing, undeclared compounds are the result of poor manufacturing practices, and it is difficult to avoid because the majority are labeled as vitamins, minerals, proteins, or other non-prescription products. However, some plant extracts should be avoided to prevent the unintentional intake of doping compounds by food supplements. Most of these extracts are used as stimulants. A good example is methylhexaneamine (Geranamine), a stimulant included in the World Anti-Doping Agency’s (WADA) prohibited list, which may be found in the *Geranium* and *Pelargonium* species known collectively as *Geraniaceae* [17]. According to WADA, there have been several examples of athletes testing positive for methylhexaneamine in recent years, notably during the Olympic Games. WADA warns that the term “methylhexaneamine” is rarely seen on dietary supplement labels, and this stimulant is often known as geranium root extract or geranium oil [18]. Professional athletes should avoid food supplements labeled as geranium root extract or geranium oil.

**Figure 2:**
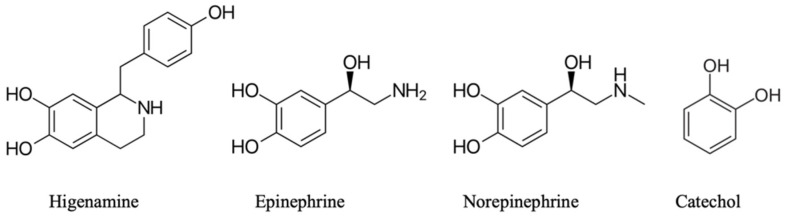
Higenamine, epinephrine, norepinephrine, and catechol structures.

Higenamine (1-[(4-hydroxyphenyl) methyl]-1,2,3,4-tetrahydroisoquinoline-6,7-diol is a benzyltetrahydroisoquinoline alkaloid whose structure is similar to epinephrine and norepinephrine (Figure 2). The compound is a natural agonist of β-adrenoreceptors. 

Despite the similarities between these structures, there are some important differences: epinephrine and norepinephrine are classical examples of catecholamines (Figure 2), whereas higenamine consists of a catechol ring attached to a piperidine ring joined to a p-hydroxybenzyl group. 

**Figure 3:**
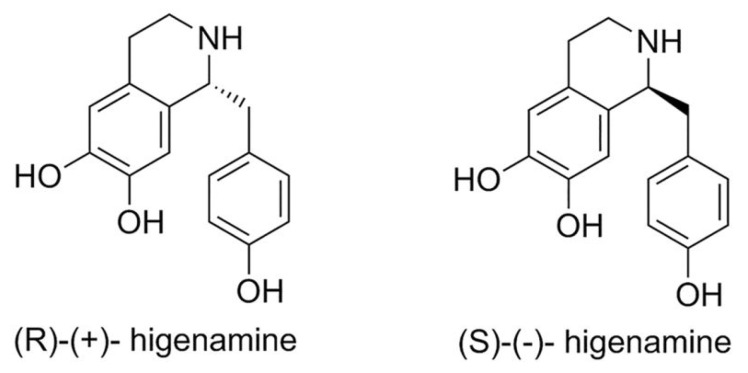
*S*-(-)-higenamine and *R*-(+)-higenamine structures.

Higenamine has an asymmetric carbon (C-1) in its structure and can exist in two conformations, *S*-(-)-higenamine and *R*-(+)-higenamine (Figure 3) [19,20,21,22]. *S*-(-)-higenamine is an important biosynthetic precursor in the biosynthesis of benzylisoquinoline alkaloids in plants [19]. 

The biosynthetic pathway that leads to *S*-(-)-higenamine originates from Pictet–Spengler condensation of dopamine and 4-hydroxyphenylacetaldehyde (4-HPAA) by norcoclaurine synthase [20,21]. Although it is expected for norcoclaurine synthase to produce only tetrahydroisoquinolines with *S*- stereochemistry [22], higenamine was isolated in a racemic form or as *R*-(+)-higenamine from several plants [23,24,25].

There are several differences between the biological activity of the two enantiomers. According to recent studies, *S*-(-)-higenamine demonstrates stronger cardiotonic properties and anti-platelet effects than *R*-(+)-higenamine [24,26]. It was also reported that *S*-(-)-higenamine shows better effects in disease that are associated with inducible nitric oxide synthase over-expression [27]. These important differences in their bioactivities lead to the development of enantioselective methods for the synthesis of the two enantiomers [22,26].

For the first time the compound was isolated from aconite roots (*Aconitum japonicum*; *Ranunculaceae*) by Kosuge in 1976 [24] and identified as *Aconitum*’s active cardiotonic compound. In Chinese and Japanese folk medicine, *Aconitum japonicum* has an important role. Its extracts are keenly used to treat fever, collapse, pain, gastroenteritis, diarrhea, edema, bronchial asthma, and various tumors [28].

In Europe and North America, higenamine is a common ingredient in dietary supplements and is used for its anti-obesity and performance-enhancing effects [29]. A study from the Netherlands reported that between 2013 and 2018, 28 of 416 supplements analyzed contained higenamine. Data suggest that higenamine ranks fifth after caffeine, synephrine, sildenafil, and icariin as an adulterant in dietary supplements [30].

Due to its structural similarities to epinephrine, higenamine interacts with β_2_ adrenoreceptors and increases cardiac output and heart rate. Feng et al.’s study showed that the heart rate accelerated within 2 min of administering higenamine [31]. Higenamine has a short half-life—about 8.0 min—and is quickly eliminated from the body. According to their findings, only 9.3% was removed by renal excretion, whereas the liver played a larger role in its elimination. A total of 94% of the compound is eliminated from the human body after 30 min [31]. 

Various studies conducted over time have described the positive inotropic and chronotropic effects of higenamine (agonist of b1 and b2 adrenergic receptors) [31,32,33,34,35]. Higenamine also has a hypotensive effect, correlated with its antagonist effect to α_1_-adrenergic receptors [36]. It also inhibits the signaling pathways of TGF-b1/Smad and plays a role in preventing fibrosis and cardiac hypertrophy [37]. The anti-inflammatory effects of higenamine are due to its suppression of interleukin-1β [38]. Investigations have also reported antioxidant activity by the reduction of reactive oxygen species and induction of heme oxygenase-1 [39,40]. The intake of higenamine is associated with enhanced glucose uptake and glucose metabolism, relaxation of the trachea and colon, and anti-thrombotic effects [41,42,43]. According to its mechanism of action, higenamine can be used for treating heart failure, erectile dysfunction, bradyarrhythmia, and arthritis [1].

Since 2017, higenamine has been included in the World Anti-Doping Agency’s (WADA) Prohibited List because of its β_2_-adrenergic properties [44]. Although the compound has demonstrated beneficial therapeutic effects on different disorders [1], no medicines currently contain higenamine. At the same time, athletes might unintentionally ingest higenamine because it is present in the phytochemical composition of many herbs included in food supplements. 

Higenamine can be found in a wide range of commercially available supplements; however, the labeling of such items may not indicate its existence or appropriately describe the amount contained. Cohen et al. discovered higenamine in 24 easily accessible supplements, most of which advertised weight loss and energy enhancement. The researchers reported that higenamine concentrations in these products were 62 ± 6.0 mg per serving [45]. Higenamine consumption can increase blood pressure and cause an irregular heartbeat [29,31]. Intravenous administration of higenamine at the dose of 22.5 µg/kg was shown to have a good degree of safety [31]. Minor and short-term adverse effects after the intake of 22.5 µg/kg of higenamine, such as dizziness, chest congestion, heart palpitations, chest congestion, and dry mouth, have been reported in human clinical studies [46,47]. A study of 48 healthy men reported that peroral higenamine intake for eight weeks did not significantly change heart rate, blood pressure, blood lipids, or liver enzyme activity [47]. However, higenamine’s toxicity is related mostly with the cardiovascular systems by enhancing aconitine-induced tachyarrhythmia and myocardial contractility [32].

Jeter et al. reported the case of a 22-year-old with paraspinal muscle rhabdomyolysis associated with the use of dietary supplements containing higenamine, but the quantity of higenamine was not declared [48]. 

WADA set criteria for higenamine as a prohibited substance, stating that analytical findings should not be reported at levels under 10.0 ng/mL [49]. Appropriate methods for detecting and quantifying higenamine in dietary supplements and biological samples have been developed to minimize the misuse of higenamine, including LC–MS/MS, UHPLC–MS/MS, and GC–MS combined with derivatization [50,51,52,53,54].

In a 2020 report from WADA, anti-doping testing figures show that higenamine use is increasing and that higenamine ranked second as a β2 agonist. Higenamine had adverse analytical findings in 26 cases [55]. The USADA (United States Anti-Doping Agency) reported different sanctions ranging from 10 to 20 months for athletes that violated anti-doping rules by testing positive for higenamine. Investigations attributed positive samples to the use of dietary supplements [56,57,58].

Although higenamine is a doping agent found in many plants and food supplements, data about higenamine in the English language are limited. Data concerning sources of higenamine and its pharmacological activity should be included in educational programs for doping prevention.

Our study aims to support doping prevention and the development of educational programs for athletes about doping sources.

## 2. Materials and Methods

**Figure 4:**
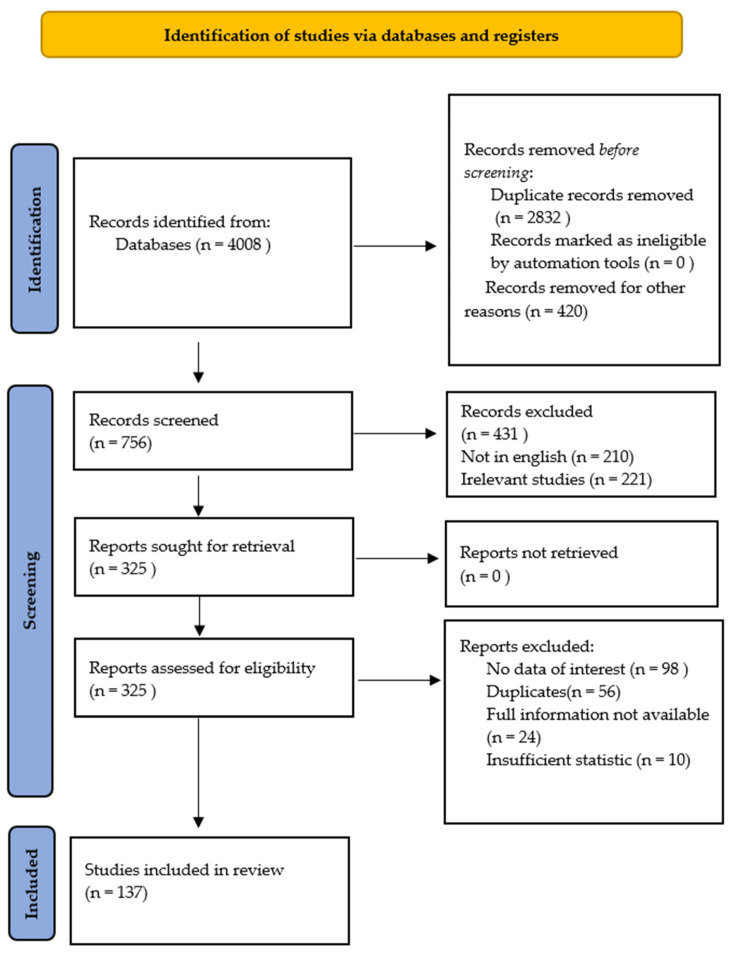
PRISMA 2020 flow diagram for new systematic reviews, which includes database and register searches only.

We searched scientific electronic databases for eligible studies, including PubMed, Google Scholar, Science Direct, and Web of Science. We followed the PRISMA guidelines for conducting systematic reviews (Figure 4) [59]. We conducted our search using the following keywords: “higenamine in dietary supplements”, “higenamine detection”, “higenamine in plants”, “ higenamine in plants detection“, “higenamine in *Nelumbo nucifera*”, “higenamine in *Nandina domestica*”, “higenamine in *Tinospora crispa*”, “higenamine in *Aconitum*”, “higenamine in *Gnetum parvifolium*”, and “higenamine in *Asarum sieboldii*”. 

We discovered other relevant sources by inspecting the references of related articles. The program Zotero was used to generate the references in this paper.

The following eligibility criteria were used in selecting articles:

- Original articles that provide information on higenamine presence in plants;
- Manuscripts proving the presence of bioactive compounds in related plants;
- Studies providing methods for identification or quantification of higenamine in plants;
- Studies involving human, mammals, or biological samples investigating the relationship between the use of higenamine-containing plants and other possible effects.

Titles, abstracts, and full texts were screened. One researcher reviewed the whole text of the preselected publications to apply eligibility criteria, while a second researcher double-checked the selections to verify that all studies were included. One author collected data and verified them with another author to avoid missing data. Only full-text original manuscripts written in English were included. Our last search was performed on 10 November 2021.

## 3. Results and Discussion

Data from our bibliographic review indicate that higenamine is found in various plants, including *Aconitum* spp. [60,61], *Tinospora crispa* *(Tinospora crispa* (L.) Hook. f. & Thomson) [62], *Nandina domestica* (*Nandina domestica* Thunb.) [63], *Nelumbo nucifera (Nelumbo nucifera* Gaertn.) [63,64,65], *Gnetum parvifolium (Gnetum parvifolium* (Warb.) W.C.Cheng) [25], *Asarum siebodii* (*Asarum sieboldii* Miq.) [60], and *Aristolochia brasiliensis (Aristolochia brasiliensis* Mart. & Zucc.) [66]. Extracts from these plants are available as food supplements. Studies of higenamine detection in plants are summarized in Table 1. The data about *Tinospora crispa* are presented in Table 2.

### 3.1. Nelumbo nucifera (Nelumbo nucifera Gaertn.)

*Nelumbo nucifera* (sacred lotus) is an aquatic rhizomatous plant of the monogeneric family *Nymphaeaceae*. *Nelumbo nucifera* has peltate, membranous leaves up to 90 cm in diameter with tiny thorns along the long petiole. The flowers are approximately 10–25 cm in diameter, have many stamens and various colors, ranging from rosy to white. The inner part of the yellow rhizome contains air pockets that enable flotation in water. Fruits are green and ovoid with black, hard seeds arranged in whorls [77,78]. Nearly all parts of the lotus plant worldwide are used as vegetables. Reports on the therapeutic properties of lotus date back to 400 B.C. and were originally documented in the book *Erya* [79]. *Nelumbo nucifera* contains a wide range of chemical compounds: alkaloids, flavonoids, glycosides, terpenoids, fatty acids, steroids, minerals, and vitamins [80]. Compounds found in lotus are associated with various pharmaceutical effects. Flavonoids have been shown to have antioxidant activity [81]. Alkaloids also show strong cytoprotective activity, primarily liensinine, neferine, and roemerine [82].

Flavonoids have antiviral, hepatoprotective, anti-obesity, anti-inflammatory, anxiolytic effects, and alkaloids effective in treating cardiovascular diseases; they also have anti-inflammatory effects, decrease blood lipids, and have melanogenesis inhibitory activities [64,83,84,85,86,87,88,89,90,91,92]. Nuciferine, (-)-lirinidine *N*-methylasimilobine, and 2-hydroxy-1-methoxy-6a,7-dehydroaporphine provide melanogenesis inhibitory activity [93]. There have been several studies investigating the phytochemistry of *Nelumbo nucifera*. It is rich in flavonoids and glycosides. It is also rich in alkaloids, including coclaurine, 4′-*N*-methyl coclaurine, nuciferine, higenamine, higenamine 4-*O*-glucoside, and nelumboferine [93,94,95,96,97]. Pei et al. detected 171 compounds from six different parts of the lotus in a recent study [69]. Higenamine was identified in the leaves and plumule.

*S*-(-)-higenamine was isolated from leaves and *R*-(+)-higenamine was isolated from the embryo of *Nelumbo nucifera* [68,87].

Lotus plumule, also known as *Plumula nelumbinis*, and Lian Zi Xin, the bitter-tasting green embryo of lotus seeds, has long been used as tea in Asia. It has been used to treat mental health problems, sleeplessness, high-temperature fevers, and cardiovascular disease [98,99]. 

Although the use of lotus extract has a rich history, randomized studies involving humans are limited.

However, there are some studies that investigated the pharmacokinetics of higenamine after the intake of lotus plumule. Yen et al. conducted a study where six human volunteers consumed 0.8 g of lotus plumule extract powder and 679.6 μg of higenamine, respectively, three times a day for three days. HPLC–MS/MS was used to determine the amount of higenamine. The authors of this study recommended avoiding the intake of herbal products containing lotus plumule by professional athletes because it would result in high urinary concentrations of higenamine and violation of anti-doping rules. [100]. In another study, volunteers were administered *Plumula nelumbinis* capsules with a 0.34 g/capsule six times a day for seven days. Urine samples were analyzed with UPLC–MS/MS [72]. The concentration of higenamine in the urine of most sample groups from both studies was higher than the limit stated in the WADA regulations [49]. Lotus plumule herbal extract intake clearly poses a risk to anti-doping violation rules. These results suggest that athletes should avoid eating foods or medicines containing *Plumula nelumbinis* during and outside of competition. 

### 3.2. Tinospora crispa (Tinospora crispa (L.) Hook. f. & Thomson)

*Tinospora crispa* (*Menispermaceae*) is a woody vine with large, heart-shaped leaves 6–12 cm long and 7–12 cm wide with small yellow or green flowers. The stems are long, fleshy, and thick, with numerous tubercles. Its petioles are 5–15 cm long [101]. *Tinospora crispa* is widely used in traditional medicine in Southeast Asia. This herb has been used as an antipyretic for treating rheumatism, diabetes, hypertension, stimulation of appetite, and maintaining good health [102]. The decoction of the stem has been used for its antipyretic, antimalarial, and anthelmintic properties, whereas the decoction of the whole plant is used in treating diabetes [103]. Fresh leaves are reportedly used on wounds [104]. Medicinal compounds are found in all parts of the plant (roots, leaves, and stems). Pharmacochemical studies indicate the presence of flavonoids and glycosides, including apigenin and genkwanin; alkaloids, including higenamine, tyramine, dihydrodiscretamin, columbamine, *N*-demethyl-*N*-formyldehydronornuciferine, *N*-formylasimilobine2-*O*-β-*D*-glucopyranoside, *N*-formylasimilobine2-*O*-beta-*D*-glucopyranosyl-(1-->2)-beta-*D*-glucopyranoside, *N*-trans-feruloyltyramine, *N*-formylnornuciferine, *N*-formylanonaine, terpenoids and others such as adenosine, uridine, and adenine [105,106,107,108,109]. Extracts from the *Tinospora crispa* stem show high antioxidant activity, which can be measured with vitamin C and butylhydroxytoluene [110,111,112]. *N*-cis-feruloyltyramine, *N* trans-feruloyltyramine, and secoisolariciresinol are responsible for antioxidant activity [111]. Borapetosides A and C from *Tinospora crispa* stem extracts correspond to anti-hypoglycemic effects [113,114,115]. Based on a recent study by Rakib et al., genkwanin has hepatoprotective components from *Tinospora crispa* [106].

Active compounds isolated from *Tinospora crispa* affect the cardiovascular system. Five active compounds were isolated by Praman et al., including higenamine, adenosine, uridine, salsolinol, and tyramine. [73]. These chemicals influenced blood pressure and heart rate in normal, anesthetized, and reserpinized rats. Adenosine and salsolinol both decreased heart rate and arterial blood pressure. Higenamine raised heart rate while lowering mean arterial blood pressure. In *Tinospora crispa*, higenamine was found in a racemic mixture of *R* and *S* enantiomers in concentrations from 0.001 to 0.3 mg/kg [62,73]. Study results have suggested that *Tinospora crispa* is a promising plant for treating many diseases. Although few human studies provide evidence of higher than recommended urinary concentrations of higenamine, our recommendation for athletes is to restrain their use of *Tinospora crispa* extracts. More comprehensive studies on the higenamine content of *Tinospora crispa* are required to understand the potential dangers of higenamine misuse for athletes.

### 3.3. Aconitum spp.

*Aconitum* is a major genus in the *Ranunculaceae* family, including over 400 species. The first use of the *Aconitum* species dates to 2000 years ago [116]. Despite many of the species in this genus being toxic, they have been used in China, Korea, Japan, and India as medical herbs, whereas the use of *Aconitum* in the United States and Europe is limited [117,118]. Plants from the *Aconitum* genus have long been used to treat heart failure and poor circulation [118]. *Aconitum carmichaelii* (*Aconitum carmichaelii* Debeaux.) is currently used in traditional Chinese medicine. Familiar derivates of *Aconitum carmichaelii* are Radix *Aconiti praeparata* and Radix *Aconiti lateralis praeparata*, called Chuanwu and Fuzi, respectively [119]. Fuzi is a processed form of the lateral root. The forms of Chuanwu and Fuzi depend on different processing approaches, including Shengfuzi, Yanfuzi, Heishunpian, and Baifupian, which were created to reduce toxicity [28]. Most phytochemical studies have focused on the plant’s roots [119]. More than 120 chemical compounds, particularly alkaloids, have been isolated from *Aconitum carmichaelii*. Diterpene alkaloids are the main class of *Aconitum* plants, and they vary from nontoxic to fatal and poisonous. They can be separated into three groups: diester-diterpenoid alkaloids (DAs), monoester-diterpenoid alkaloids (MAs), and unesterified diterpenoid alkaloids (UAs). Among these alkaloids are also saponins, glycosides, and flavonoids. Diester-diterpenoid alkaloids such as hypaconitine, aconitine, and mesaconitine are responsible for toxicity and can induce arrhythmia or cardiac depression [8]. During processing methods such as decoction and steaming, diester-diterpenoid alkaloids hydrolyze in less toxic monoester-diterpenoid alkaloids so *Aconitum’s* poisonous effect decreases significantly. Many new processing methods for *Aconitum* have been developed in recent years [119,120]. Decoctions are often combined with dried ginger, licorice, and ginseng [118,121]. Examples of these decoction combinations are Sini and Shenfu. New technologies enable *Aconitum* to be used as a safe plant. Alkaloids from *Aconitum* demonstrate analgesic, cardiovascular, anti-inflammatory, antioxidant, and anti-tumor activities [122]. 

Higenamine was discovered as a cardiotonic compound in 1976 [24]. Coryneine and salsolinol were also extracted from Radix *Lateralis Preparata* [74]. Higenamine was also detected in *Aconitum kusnezoffii (Aconitum kusnezoffii* Reichb) and *Aconitum napiforme* (*Aconitum napiforme* H.Lev. & Vaniot) roots. *R*-(+)-higenamine in *Aconitum* preparations is ten times less than in the embryo of *Nelumbo nucifera*, [61]. icELISA and HPLC were used to identify *S*-(-)-higenamine in *Aconitum carmichaelii* roots. A study by Chung et al. showed a different concentration of *R*-(+)-higenamine in the processed and unprocessed roots of *Aconitum carmichaelii* at 12.2 and 18.3 μg/g [61]. Since then, higenamine has been detected in the decoctions Baifupian and Heishunpian in similar amounts ranging from 2.31 to 3.18 μg/g. [70]. Nowadays, there are many processed products of *Aconitum*. For example, in the Chinese Pharmacopoeia 2015 around 50 herbal mixtures containing *Aconitum* are described [119]. Because these products are produced with different extraction methods the concentrations of higenamine might significantly vary. The safety of each *Aconitum* preparation must be evaluated before intake by professional athletes [61,71].

### 3.4. Nandina domestica (Nandina domestica Thunb.)

*Nandina domestica* is a plant from the *Berberidaceae* family. In Chinese traditional medicine, roots, leaves, stems, and fruits are primarily used to treat coughing and asthma [123]. Phytochemical analysis has recently revealed that major compounds from *Nandina domestica* are alkaloids [123,124,125]. Flavonoids and lignans are also present [126]. Nantenine is an alkaloid that mostly contributes to the pharmacological effectivity of *Nandina domestica* [42]. Higenamine is associated with tracheal relaxation from *Nandina domestica* through the stimulation of β adrenoreceptors [75,127]. *S*-(-)-higenamine was detected in the leaf and fruit by icELISA and HPLC in the dry weight concentrations of 56.30 and 14.02 μg/g, respectively [60]. Higenamine was also isolated as a racemic mixture [128]. In 2017, Okano et al. used LC–MS/MS for detecting higenamine and coclaurine in urine after using throat lozenges containing *Nandina domestica* fruit. The urinary concentration of higenamine and coclaurine grew but did not exceed the criteria set by WADA [76]. Therefore, not every product containing *Nandina domestica* poses a risk to athletes when following the label-recommended dosage. However, excessive, and prolonged intake must also be considered.

### 3.5. Gnetum parvifolium (Gnetum parvifolium (Warb.) W.C.Cheng)

*Gnetum parvifolium* is a green woody vine from the Gnetaceae family. Phytochemical studies have revealed that it is rich in stilbenoids and flavonoids [129,130]. The roots and stems are traditionally used to treat rheumatism, chronic bronchitis, respiratory infections, and traumatic injuries [129,130,131]. Stilbenoids from the lianas of the plant have anti-inflammatory activity [129]. Resveratrol isorhapontigenin, piceatannol, and rhaponiticin have been identified as xanthine oxidase inhibitors [131]. Higenamine was first isolated from the ethanolic extract of lianas in 1980, then again in 1998 as a racemic mixture of *S*-(-)-higenamine and *R*-(+)-higenamine [25]. There are no data for the exact concentrations of higenamine in *Gnetum parvifolium.*

### 3.6. Asarum sieboldii (Asarum sieboldii Miq.)

*Asarum sieboldii* from the Aristolochiaceae family is often used in Japan, Korea, and China [132]. It has been officially classified in the Chinese Pharmacopoeia for its analgesic and antitussive effects [133]. Essential oils such as limonene, asarylketone, cineol, safrole, lignans, and alkaloids such as higenamine are the main compounds of the plant. There is evidence for the anti-inflammatory [134], anti-bacterial [135], anti-fungal [132], and anti-cancer activity [136] effects of *Asarum sieboldii*. Bioactive compounds of Asari radix, including methyl eugenol, sesamin, asarinin echinacoside, vanillic acid, and kakuol, are useful in treating allergies [137,138,139]. Xixin (Asari Radix et Rhizoma) can be radix and rhizoma from *Asarum sieboldii* or *Asarum heterotropoides* (*Asarum heterotropoides* F. Schmidt) [140]. Kosuge et al. isolated higenamine as a racemic mixture from *Asarum heterotropoides* and demonstrated how it is responsible for antitussive effects [141]. *S*-(-)-higenamine was also isolated from *Asarum sieboldii* roots in the dry weight concentrations of 23.08 µg/g with HPLC and 31.07 µg/g in dry weight with icELISA [60]. Asari Radix et Rhizoma is part of the recipe for Mahuang Fuzi Xixin [142], which contains Ephedrae Herba (Ma Huang), Radix Aconiti Lateralis Preparata (Fu Zi), Asarum Heterotropoides, or Asarum sieboldii (Xi Xin) and is used to treat asthma in traditional Chinese medicine [143]. All plants potentially contain performance-enhancing effects, and two of them contain higenamine. Combining such products can lead to higher amounts of higenamine and risks unintentional doping.

### 3.7. Risk Assessment of Intake Higenamine-Containing Plants

According to our research, the concentration of higenamine in lotus plumule was higher than in other plants. Investigations into the pharmacokinetics of lotus plumule capsules regulated with good manifesting practice and commercially used powder showed that both pose risks of unintentional doping [72,100]. Yen et al. investigated the total amount of alkaloids in six herbal extract products of Plumule Nelumbis and five crude lotus plumule. The concentration of higenamine varied from 263.9 to 969.5 μg/g. Different preparation methods lead to changes in higenamine concentrations. Furthermore, samples analyzed from six individuals show significant differences in higenamine amounts [100]. These results relate to UGT1A9 (UDP glucuronosyltransferase family 1 member A9), which is responsible for higenamine glucuronidation [144]. *Nandina domestica* (leaf) also has high concentrations. A total of 56.30 μg/g in dry weight was detected by icELISA. Nuntawong et al. compared two methods, icELISA and HPLC, where the higenamine concentrations detected with HPLC were lower (34.69 μg/g in dry weight) [60]. The same study showed that candy containing *Nandina domestica* does not contain higenamine and that urinary samples collected from the consumption of throat lozenges containing *Nandina domestica* fruit contained 0.2−0.4 ng/mL of higenamine. *Nandina domestica* fruit contains less higenamine than the leaves [60], and pharmacokinetic studies on *Nandina domestica* leaf-based products are missing. Considering that *Nandina domestica* leaf is also used to treat cough and asthma, we believe that more studies are needed to understand its safety and risk factors fully. Studies on *Nandina domestica* and higenamine’s effects on tracheal relaxation provide more reasons to restrain from the use of Nandina domestica products [75]. Although there are low concentrations of higenamine in *Tinospora crispa,* studies on crude stem extracts demonstrated adverse effects on blood pressure and left atrium contraction in rats, which correlated with higenamine presence [62,73]. More studies on the effects of higenamine, including anti-microbial effects from *Aristolochia brasiliensis*, hypolipidemic effects from *Nelumbo nucifera* in mammals, and various matrices are summarized in Table 2. Since higenamine’s effect on metabolism is unclear, UGT1A9 can vary by individual. Different extraction methods can lead to different concentrations of alkaloids. Some decoctions such as Mahuang Fuzi Xixin combine plants that contain higenamine. Before more comprehensive studies are conducted to ensure the safety of the aforementioned plants, athletes should restrict their use of any products containing *Nelumbo nucifera, Nandina domestica, Tinospora crispa, Aconitum* spp., *Aristolochia brasiliensis, Asarum sieboldii*, *Asarum heterotropoides,* and *Gnetum parvifolium.*

## 4. Conclusions

Unintentional doping has become a serious and important scientific and social task in the previous two decades. The primary sources of unintentional doping are food supplements that contain prohibited compounds, contaminated food supplements, and some plant extracts. However, unintentional doping is preventable.

The key points of this prevention are defining the sources of unintentional doping, educating athletes about the sources of unintentional doping, and helping athletes analyze and control which food supplements are included in their diet.

Higenamine is a good example of a pharmacologically active compound included in WADA’s prohibited list. It is also found in many plant extracts used by humanity for a long period of time and food supplements. The labeling of most commercial products is unclear regarding the higenamine content of a specific plant or extract. *Nelumbo nucifera, Nandina domestica, Tinospora crispa, Aconitum* spp., *Aristolochia brasiliensis, Asarum sieboldii*, *Asarum heterotropoides*, and *Gnetum parvifolium* are used for various allergic diseases, rheumatism, asthma, diabetes, hypertension, etc. Considering that plant species are receiving more attention for treatments worldwide and the rising trend of plant-based dietary supplements, it is important to include all plants containing higenamine in educational programs for athletes.

Our recommendation to athletes is to restrain their use of those plants. More comprehensive studies evaluating the risks and benefits of using certain reported plants would be valuable.

Knowledge about herbal medicine components and further investigation into plants is important so that athletes can be confident that herbal medicines are safe to use.

## Figures and Tables

**Table 1:** Methods for identification and quantification of higenamine in plant extracts.

Plant Name	Plant Part	Investigation Methods	Main Results	Reference
*Nelumbo nucifera*	dried leaf and seed	QuEChERS LC–MS/MS	Higenamine was detected in dried lotus leaf, dried lotus seeds at a concentration of 9667.6 and 1183.8 μg/kg, respectively.	[64]
*Nelumbo nucifera*	plumule	UPLC–DAD	21 alkaloids were identified, including higenamine.	[67]
*Nelumbo nucifera*	root of embryo	High-Performance Liquid Chromatography with a Fluorescent Chiral Tagging Reagent	*R*-(+)-higenamine in embryo of *N. nucifera* was 93.0 ± 2.42 mg/100 g	[68]
*Nelumbo nucifera*	seed, leaf stamen, plumule, receptacle, and rhizome node	UPLC and QToF-MS	Higenamine was detected in leaves and plumule	[69]
*Aconitum japonicum*	root	Column chromatography	Higenamine was isolated from *Aconitum Japonicum* thumb.	[24]
		icELISA and HPLC.	Higenamine amounts detected:	[60]
*Aconitum carmichaelii* (roots)	roots		2.58 × 10^−1^–3.04 μg/g dry wt	
*Nandina domestica* (fruit)	fruit		12.21 μg/g dry wt	
*Nandina domestica* (leaf)	leaf		56.30 μg/g dry wt	
*Asarum siebodii* (root)	root		31.07 μg/g dry wt	
*Evodia rutaecarpa* (fruit)	fruit		6.48 μg/g dry wt	
*Aconitum kusnezoffii* and *Aconitum napiforme*	root	HPLC	Aconitum extracts contained 8–19 μg/g of higenamine	[61]
*Aconitum carmichaelii (Radix Aconiti Lateralis Preparata)*	root	LC coupled with MS/MS	Concentration of higenamine in different decoctions were: Bifupian 2.31 ± 0.11μg/g Heishunpian 3.03 ± 0.15 μg/g	[70]
*Aconitum carmichaelii (Radix Aconiti Lateralis Preparata)*	root	LC–MS/MS	10 of the identified compounds, including quercetin, pseudoephedrine, ephedrine, *β*-asarone, methylephedrine, *α*-linolenic acid, cathine, ferulic acid, nardosinone, and higenamine accounted for most of the beneficial effects of Mahuang Fuzi Xixin in allergic rhinitis	[71]
*Gnetum parvifolium*	lianas	NMR	5 alkaloids were isolated, including higenamine	[25]

**Table 2:** Studies of higenamine in human/mammals in various matrices.

Study	Aim	Main Results	Ref.
The risk of adverse higenamine analytical findings following oral administration of *Plumula nelumbinis* capsules	Detection of higenamine in urine samples	The concentration of higenamine in most urine sample groups exceeded the limit specified by WADA.	[72]
Crude extract and purified components isolated from the stems of *Tinospora crispa* exhibit positive inotropic effects on the isolated left atrium of rats	Investigation into the mechanism of *Tinospora crispa* bioactive compounds.	Higenamine increased the left atrium’s contraction force.	[62]
Hypotensive and cardio-chronotropic constituents of *Tinospora crispa* and mechanisms of action on the cardiovascular system of anesthetized rats	Identifying the active components in *Tinospora crispa* extracts and investigating the mechanisms of action on blood pressure and heart rate in anesthetized rats.	Salsolinol, tyramine, and higenamine acted via the adrenoreceptors, whereas uridine and adenosine acted via purinergic adenosine A2 and P2 receptors to decrease blood pressure.	[73]
Counter effects of higenamine and coryneine, components of Aconite root, on acetylcholine release from motor nerve terminal in mice	Detection of higenamine counteraction on acetylcholine release.	Higenamine increases Ach release by activating β-adrenoreceptors. Coryneine depresses Ach release by acting on the motor nerve terminal.	[74]
β_2_ -adrenoceptor-mediated tracheal relaxation induced by higenamine from *Nandina domestica* Thunberg	Identifying active components and mechanism of action on tracheal relaxation	Higenamine is major constituent of *Nandina domestica* crude extract, which likely induces tracheal relaxation by stimulating β_2_ adrenoceptors.	[42]
Biphasic tracheal relaxation induced by higenamine and nantenine from *Nandina domestica* Thunberg	Comparing the effects of crude extract, higenamine, and nantenine	Higenamine relaxes the trachea by β-adrenoceptor stimulation.	[75]
Determining higenamine and coclaurine levels in human urine after the administration of a throat lozenge containing *Nandina domestica* fruit	Investigation of throat lozenge components and developing a mass-spectrometry method for quantifying higenamine and coclaurine in human urine.	The maximum concentrations of higenamine and coclaurine were 0.2−0.4 and 0.3−1.0 ng/mL. They did not exceed WADA’s criteria.	[76]
Evaluation of anti-microbacterial activity of higenamine using *Galleria mellonella* as an in vivo infection model	Investigation of anti-microbacterial effects of higenamine from *Aristolochia brasiliensis*	Higenamine was isolated from *Aristolochia brasiliensis* and showed anti-microbacterial activity.	[66]
A triple combination strategy of UHPLC–MSn, hypolipidemic activity, and transcriptome sequencing to unveil the hypolipidemic mechanisms of *Nelumbo nucifera* alkaloids	Screening of *Nelumbo nucifera* alkaloids for potential hyperlipidemic effect.	35 compounds were found in *N. nucifera* alkaloid extraction. Liensinine, higenamine, *N*-norarmepavine nuciferine, *N*-nornuciferine, *O*-nornuciferine, coclaurine and armepavine showed significant effects on hyperlipidemia.	[63]

## Data Availability

Not applicable.
